# Associations of proteomic age with mortality and incident chronic diseases in the European Prospective Investigation into Cancer and Nutrition (EPIC)

**DOI:** 10.21203/rs.3.rs-7087230/v1

**Published:** 2025-07-15

**Authors:** Oliver Robinson, Han Xiao, Jan Homann, Vivian Viallon, Pietro Ferrari, José M. Huerta, Ana Jiménez Zabala, Rudolf Kaaks, Verena A Katzke, Claudia Langenberg, ChungHo E. Lau, Lefkos Middleton, N. Charlotte Onland-Moret, Salvatore Panico, Anna Prizment, Fulvio Ricceri, María-José Sánchez, Karl Smith Byrne, Paolo Vineis, W .Monique Verschuren, Roel Vermeulen, Shuo Wang, Nick Wareham, Christina M. Lill, Elio Riboli, Marc J. Gunter

**Affiliations:** 1 Department of Epidemiology and Biostatistics, School of Public Health, Imperial College London, UK; 2 Ageing Epidemiology Research (AGE) Unit, School of Public Health, Imperial College London, UK; 3 Institute of Epidemiology and Social Medicine, University of Münster, Germany; 4 International Agency for Research on Cancer, France; 5 Department of Epidemiology, Murcia Regional Health Council-IMIB, 30007 Murcia, Spain.; 6 Centro de Investigación Biomédica en Red de Epidemiología y Salud Pública (CIBERESP), 28029 Madrid, Spain.; 7 Ministry of Health of the Basque Government, Sub Directorate for Public Health and Addictions of Gipuzkoa, 20013 San Sebastian, Spain; 8 Biodonostia Health Research Institute, Epidemiology of Chronic and Communicable Diseases Group, 20014 San Sebastián, Spain; 9 Division of Cancer Epidemiology, German Cancer Research Center (DKFZ), Heidelberg, Germany; 10 Precision Healthcare University Research Institute, Queen Mary University of London, UK; 11 MRC Epidemiology Unit, University of Cambridge, UK; 12 Julius Center for Health Sciences and Primary Care, University Medical Center Utrecht, Utrecht University, Utrecht, the Netherlands; 13 Federico II University, Naples, Italy; 14 Department of Laboratory Medicine and Pathology, University of Minnesota, Minnesota, USA; 15 Centre for Biostatistics, Epidemiology, and Public Health (C-BEPH), Department of Clinical and Biological Sciences, University of Turin, Italy; 16 Escuela Andaluza de Salud Pública (EASP), 18011 Granada, Spain; 17 Instituto de Investigación Biosanitaria ibs.GRANADA, 18012 Granada, Spain; 18 Centro de Investigación Biomédica en Red de Epidemiología y Salud Pública (CIBERESP); 19 Cancer Epidemiology Unit, University of Oxford, UK; 20 National Institute for Public Health and the Environment, Bilthoven, the Netherlands; 21 Institute for Risk Assessment Sciences at Utrecht University.

**Keywords:** Aging, biological age, biological clocks, neurodegeneration, cancer, cardiovascular disease, diabetes, risk factors, risk prediction, proteomics, SomaLogic, aptamers

## Abstract

Assessment of biological ageing using proteomic clocks may enhance risk prediction and elucidate the molecular links between ageing and chronic diseases. Within a pre-diagnostic cohort of 17,473 Europeans with up to 28 years of follow-up, we examined associations of plasma SomaScan-based proteomic clocks, including organ-specific clocks, with 24 incident chronic diseases, all-cause mortality, and lifestyle risk factors. Global proteomic age gap (a composite biological age acceleration score combining previously published clocks) showed the strongest positive association of all tested clocks with all-cause mortality. Accelerated proteomic ageing was significantly associated with smoking, alcohol consumption, physical inactivity, and higher risk of cardiovascular diseases, dementia, and liver, upper aero-digestive tract, lung, and kidney cancers. Some organ-specific cancers were more strongly associated with their respective organ-specific age gaps. Mortality prediction by proteomic clocks was comparable in performance to classical lifestyle risk factors. In summary, proteomic clocks appear promising biomarkers of generalized age-related disease risk.

## Introduction

Although age has long been recognized as the most important risk factor for most chronic diseases, it has only recently become a focus of risk factor epidemiology and interventional research, with the recognition that ageing rates may be modifiable ^1^. Geroscience proposes that biological ageing, the cellular and molecular changes associated with ageing, underlines the risk of multiple age-related diseases, and through targeting these processes, health in later life can be improved ^2^. This is particularly relevant given the ageing population and the challenge of closing the health span and lifespan gap ^3^. To understand the role biological ageing may play in disease risk and the causes of variation in ageing rates, quantitative markers of biological age are required that are applicable at scale in human studies.

Since the introduction of the DNA methylation-based clock by Horvath ^4^, multiple studies have developed biological age estimators (“clocks”). They are built from multivariate models trained on chronological age using high-throughput molecular profiling or “omics” data ^5^. The clocks provide an average molecular profile at a given chronological age based on the reference training population. The difference between the clock age and chronological age (the “age gap” or “age acceleration”) may then be interpreted as the biological age component of the clock prediction ^5^. Proteomics in plasma provides many advantages over other omics for biological age assessment, including greater interpretability and standardized arrays. Circulating proteins also provide systematic assessment of tissues throughout the body ^6^. In the UK Biobank, clocks have been developed using the Olink Proximity Extension Assay -based assay of around 3,000 proteins ^7–9^, that predict age across external ethnically distinct populations ^7^ and multiple incident diseases and mortality ^7–9^. In parallel, multiple clocks have been developed using aptamer-based Somascan assays, with assay coverage ranging from around 1,300 ^10^ to 5000 proteins ^11–13^ . These clocks have been trained in mainly healthy, populations of differing age ranges and geographical location, and shown to predict mortality ^12–14^ and/or age-related phenotypes ^14,15^. Furthermore, Oh et al. ^13^ developed organ specific proteomics clocks, trained on proteins putatively annotated to their organ sources.

While the Somascan-based clocks appear promising for the assessment of biological age, they remain to be widely tested in independent populations against multiple incident diseases, and the influence of different training populations on clock performance is unclear. In line with the Geroscience hypothesis, proteomic age gaps should have stronger associations with diseases that are observed to be more closely related to age itself. Furthermore, it is important for their potential use as surrogate endpoints in lifestyle interventions, that proteomic age clocks are sensitive to risk factors of non-communicable diseases. Within the large-scale, European Prospective Investigation into Cancer and Nutrition (EPIC) cohort, we sought to comprehensively assess associations of five existing proteomic clocks, a novel “Global” proteomic age clock, and organ specific clocks with risk of mortality, cardiometabolic diseases, neurodegenerative diseases and cancers. We compared the strengths of associations across different clocks and chronic diseases. Finally, we tested associations of clocks with lifestyle risk factors, including a healthy lifestyle index.

## Results

### Study Population

**Figure 1:**
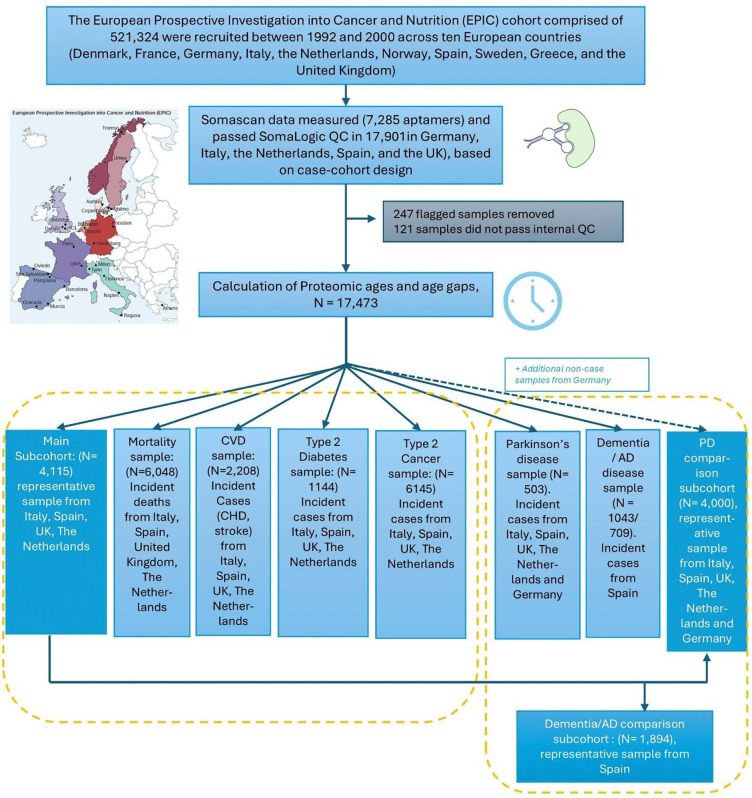
Study design and sampling flow chart. AD: Alzheimer’s Disease, PD: Parkinson’s Disease, CVD: Cardiovascular disease, CHD Coronary Heart Disease

This study analyzed baseline plasma samples from 17,815 subjects drawn from the broader EPIC cohort using a case-cohort design for multiple disease outcomes (Figure 1). The random subcohort sample set, broadly representative of the wider EPIC cohort, served as the control set for different case samples sets and included naturally occurring incident diseases and deaths. The subcohort included 4,115 subjects from Italy, Spain, the Netherlands and the UK (Supplementary table S1). Mean age in the subcohort was 51.4 (standard deviation (SD): 8.6) years and 62% were female. Mean BMI was 26.9 (4.3), 25% were smokers and 14% had a university level education. During a mean follow-up time of 17.4 (2.9) years, 393 (9.6%) deaths occurred in the subcohort. Separate case samples included 6,048 incident cases for mortality, 6,146 for cancers (17 cancer types), 2,208 for cardiovascular disease (coronary heart disease and stroke), 1,114 for type 2 diabetes, and 1,546 for neurodegenerative diseases (all-cause dementia, Alzheimer’s disease (AD) and Parkinson’s disease [PD]). The neurodegenerative disease arm of this study involved a slightly different composition of participating centers per disease and comparison subcohorts (Figure 1). Population characteristics of all the cases sample sets, and the comparison subcohorts used for the neurodegenerative disease analyses, are presented in Supplementary tables S2 – S9.

### Proteomic age calculation

**Figure 2:**
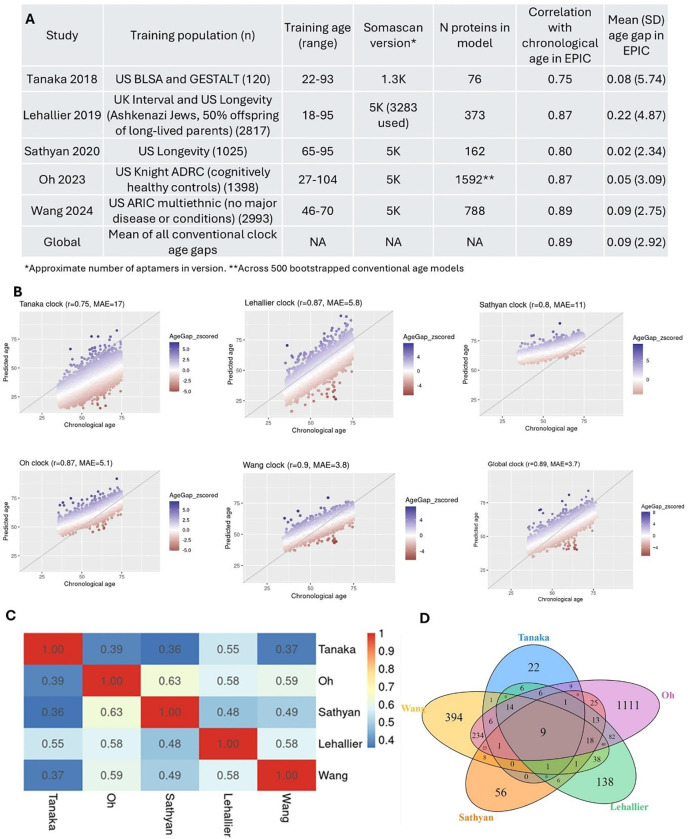
Overview of proteomic clocks. A: Table of clock characteristics. B: Scatterplots of predicted versus chronological age for all clocks used, showing Pearson’s correlations and mean absolute error (MAE). C: Correlation heatmap of proteomic age gaps. D: Venn diagram showing overlap of proteins included in each clock

The characteristics of five “conventional” clocks included in the study are shown in Figure 2A. The clocks were trained previously in other populations and are referred to as conventional as they were developed using all available proteins, as opposed to preselecting proteins as for organ-specific clocks. The clock-predicted ages were all strongly correlated with chronological age in the full EPIC sample, ranging from r = 0.75 for the Tanaka clock to r = 0.89 for the Wang clock (Figure 2A and B). The age gap standard deviations ranged from 5.7 years for the Tanaka clock to 2.3 years for the Sathyan clock (Figure 2A). Age gaps were moderately correlated across the clocks, ranging from 0.37 for Tanaka and Wang age gaps to 0.63 for Sathyan and Oh age gaps (Figure 2C). Only nine proteins (ISLR2, EGFR, PTN, ADAMTS5, GDF15, MMP12, CDON, LPO, and RSPO4, Figure 2D) were shared as predictors across the clocks. To provide a single proteomic age gap estimation, we averaged the proteomic ages and age gaps across the clocks to develop a “Global proteomic age”. The correlation of Global proteomic age with chronological age was 0.89.

### Associations of conventional proteomic age gaps with mortality and incident diseases

**Figure 3:**
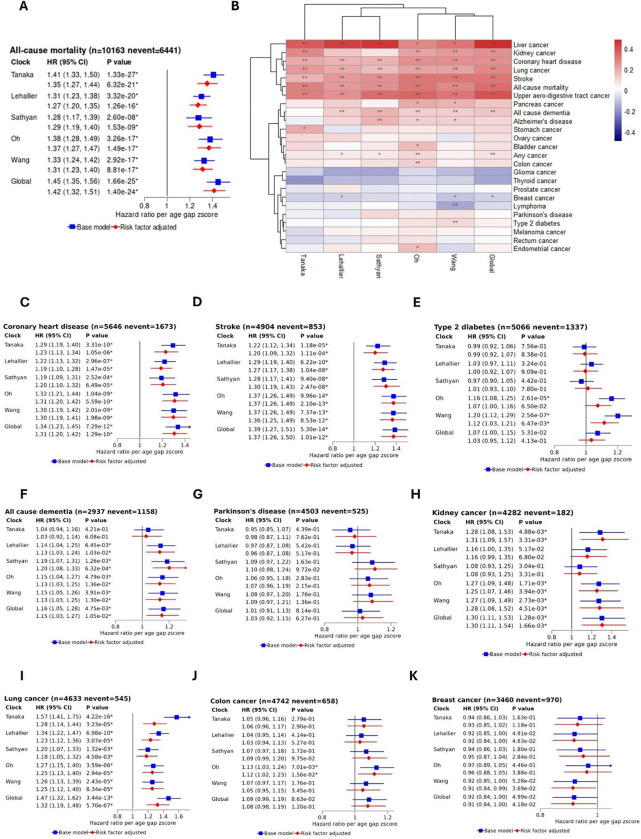
Associations of conventional proteomic age gaps with mortality and incident diseases. A: Hazard ratios per conventional proteomic age gap z-score with all -cause mortality. B: Clustered heatmap showing risk factor-adjusted associations (log hazards per age gap z-score) for all -cause mortality and 25 incident diseases. * p<0.05; ** FDR-adjusted p <0.05. C-E: Hazard ratios per conventional proteomic age gap z-score with cardiometabolic diseases. F-G: Hazard ratios per conventional proteomic age gap z-score with neurodegenerative diseases. H-K: Hazard ratios per conventional proteomic age gap z-score with common cancers, including kidney, lung, colon and breast. All models stratified by study centre, sex, and five-year age group. Risk factor adjusted model additionally adjusted for education level, smoking status, alcohol consumption, BMI, healthy diet score and physical activity. All error bars show 95% confidence intervals.

All conventional age gaps were significantly associated with all-cause mortality after FDR correction and estimates were similar in both the base model (stratified by study centre, sex, and five-year age group) and the risk factor-adjusted model (additionally adjusted for smoking status, alcohol consumption, BMI, healthy diet score and physical activity). Estimates across the five published conventional clocks were similar, ranging from a hazard ratio (HR) of 1.27 (95% confidence interval (CI): 1.20–1.35) for the Lehallier age gap z-score (i.e. one SD in proteomic age gap, equivalent to around 5 years age gap for this clock) to an HR of 1.37 (95% CI: 1.27–1.47) for the Oh age gap z-score (approximately 3 yrs age gap) in the risk factor adjusted models. Notably, a stronger association with all-cause mortality was observed for the Global proteomic clock, with a HR of 1.42 (95% CI: 1.32–1.51) per age gap z-score (approximately 3 yrs age gap) in the risk factor adjusted model (Figure 3A). In terms of increased mortality risk per year of age gap, estimates ranged from 5% for the Tanaka clock to a 13% greater risk for the Global proteomic clock (Figure S1)

After false discover rate (FDR) correction, all clocks were positively associated with risk of coronary heart disease (Figure 3C), stroke (Figure 3D), lung cancer (Figure 3I), and upper aero-digestive tract cancer in risk factor adjusted models (Figure 3B). Five clocks were associated with all-cause dementia (Figure 3F) and four clocks were associated with liver and kidney cancer (Figure 3H). Again, for these outcomes, often slightly larger effect sizes were observed for the Global proteomic age gap compared to the conventional clocks, with HRs of 1.31 (95% CI: 1.20–1.42) for coronary heart disease, 1.37 (95% CI: 1.26–1.50) for stroke, 1.32 (95% CI: 1.19–1.48) for lung cancer, 1.50 (95% CI: 1.29–1.74) for UDAT cancer, 1.63 (95% CI: 1.25–2.12) for liver cancer, 1.30 (95% CI: 1.11–1.54) for kidney cancer, and 1.15 (95% CI: 1.03–1.27) for all cause dementia, per age gap z-score in risk factor adjusted models. For Alzheimer’s disease, all but the Tanaka clock showed positive directions of effect, with only the association with the Sathyan clock (HR: 1.20 (95% CI: 1.07–1.34) reaching FDR-corrected significance

Both the Oh and Global proteomic clocks were significantly associated with the incidence of any cancer. For colon, pancreas, stomach and bladder cancers, generally positive directions of association were observed across the clocks, with only the association between the Oh clock and colon cancer (Figure 3J) significant after FDR correction. For glioma, thyroid, prostate, breast and lymphoma cancers generally inverse directions of effect were observed across the clocks. Associations with type 2 diabetes were inconsistent across clocks and greater differences between the base and risk factor adjusted models were observed. Associations for Parkinson’s disease (Figure 3G), endometrial cancer, rectum cancer and melanoma were generally inconsistent and non-significant across the clocks. Generally, a similar pattern of associations with disease was observed across the clocks (Figure 3B).

### Comparison of strengths of associations with Global proteomic age across disease endpoints

**Figure 4:**
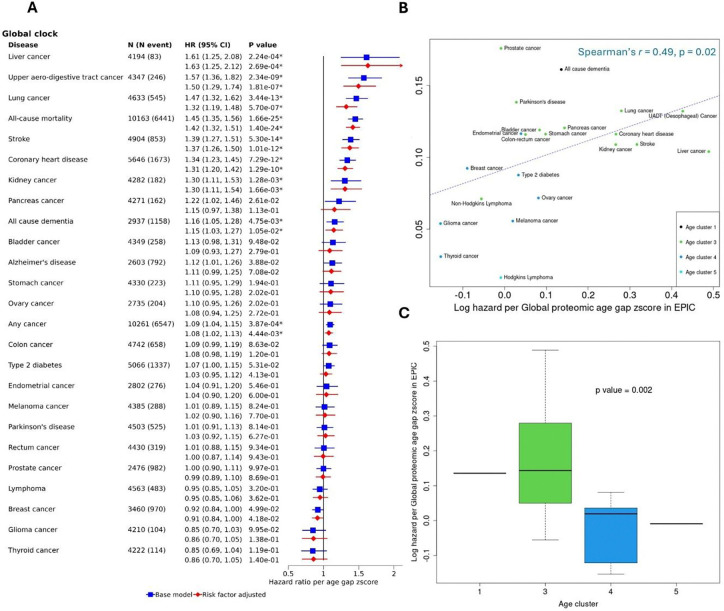
Comparison of strengths of associations with Global proteomic age across disease endpoints. A: Hazard ratios per Global proteomic age gap z-score with all -cause mortality and 25 incident diseases. All models stratified by study centre, sex, and five-year age group. Risk factor adjusted model additionally adjusted for education level, smoking status, alcohol consumption, BMI, healthy diet score and physical activity. All error bars show 95% confidence intervals. B: Scatterplot of log hazards per Consensus proteomic age gap z-score (risk factor adjusted model) against rate of disease incidence increase with age from UK National health records for 22 diseases. Extracted from Kuan et al. Sci Rep. 2021 Feb 3;11(1):2938 X. Correlation shows Spearman’s correlation. Disease points are labelled and colored by age cluster as reported by Kuan et al. C: Boxplots showing log hazards per Consensus proteomic age gap z-score (risk factor adjusted model) within each age cluster. P value calculated from t-test comparing log hazards for cluster 3 diseases versus cluster 4 diseases.

Figure 4A shows associations between the Global proteomic age gap and all disease endpoints. The largest effect size was observed for liver cancer, followed by upper areodigestive tract cancer and then all-cause mortality. Nine out of 25 (36%) mortality and disease endpoints were positively associated with the Global age gap. Similar patterns of associations were observed for the published conventional proteomic age gaps (Supplementary figures S2-S6). To explore the heterogeneity in disease associations, we compared the strength of age gap associations observed in the EPIC study with associations with (chronological) age estimated from the UK electronic health records by Kuan et al.^16^. Among 22 disease endpoints that could be matched to diseases reported by Kuan et al. ^16^ we observed a moderate positive correlation (Spearman’s rho = 0.49, p= 0.02) between the log hazards per Global proteomic gap z-score and the UK rates of disease incidence increase with age (Figure 4B). Positive directions of association with the Global proteomic age gap z-score were observed for almost all diseases in age clusters 1 and 3 (as defined by Kuan at al.^16^ , Figure 4B and 4C), while generally weaker or even negative directions of effect were observed for diseases in age clusters 4 and 5.

In sensitivity analyses, we examined associations with the Global proteomic age gap and disease endpoints restricting to events that occurred only after two years (Supplementary figure S7) and after five years (Supplementary figure S8) since recruitment. Estimates were similar except for some attenuation in the association with kidney cancer. We also restricted analyses to never smokers only (Supplementary figures S9), again finding similar estimates, except for lung cancer for which we had far fewer cases.

### Associations of risk factors with proteomic age

**Fig 5:**
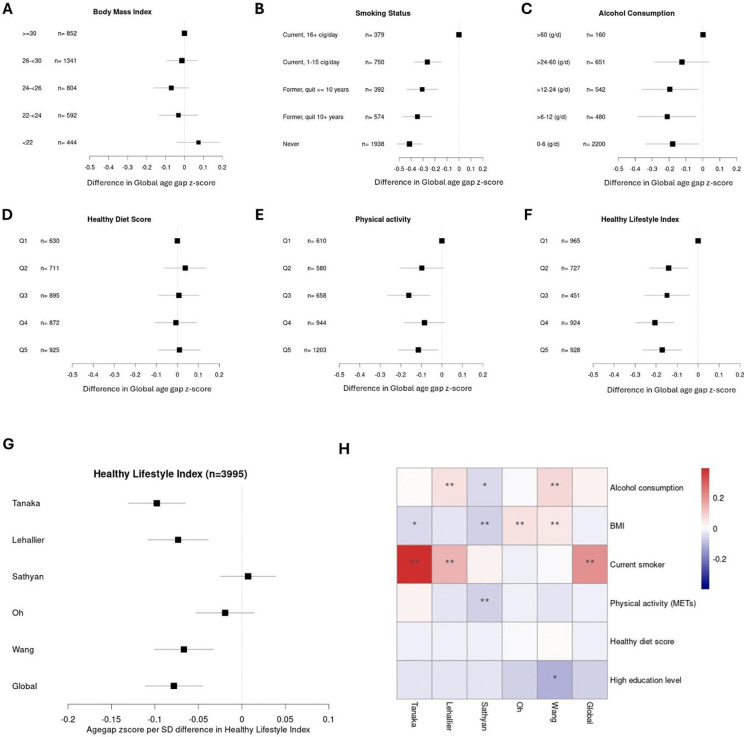
Associations of risk factors with conventional proteomic age gaps in the main subcohort. A-F: Risk factor and Healthy lifestyle index associations with the consensus age gap z scores. Adjusted for age, sex and study centre. Reference category is always the least healthy category. Q= quartiles. G: Associations between continuous healthy lifestyle index z-score and conventional age gap z scores. Adjusted for age, sex, and study centre, H: Heatmap showing associations of risk and protective factors with conventional age gap z-scores. Estimates from mutually adjusted model including all factors shown plus for age, sex and study centre. Continuous factors (alcohol intake, BMI and healthy diet score) are scaled to facilitate comparison. Reference groups for current smoker are non/former smoker. High education level refers to equivalent or university degree or higher and the reference category is education up to technical or secondary school. All error bars show 95% confidence intervals. N=3995

In analyses within the subcohort, we found that compared to those smoking 16 cigarettes or more per day, never-smokers had on average 0.41 SDs lower (95% CI: −0.52, −0.31, Figure 5B) Global proteomic age gap. Drinking only lightly or not at all (less than 6 g ethanol/day) was associated with on average 0.18 SDs lower (95% CI: −0.33, −0.02, Figure 5C) Global age gap, while the most physically active (top quintile) had on average 0.11 SDs lower (95% CI: −0.22, −0.02, Figure 5E) Global proteomic age gap compared to the most inactive (first quintile). Those in the highest quintile of the healthy lifestyle index (HLI), a 20-point scale based on diet quality, physical activity levels, smoking history, alcohol consumption and BMI, had on average 0.17 SDs lower (95% CI: −0.26, −0.08) Global proteomic age gap compared to lowest quintile (Figure 5F). No associations were observed with BMI (Figure 5A) and diet (Figure 5D).

The HLI was inversely associated with age gap z-score for all conventional proteomic clocks, except the Oh and Sathyan clocks (Figure 5G). These differences are likely driven by inconsistencies across the clocks in their association with individual non-communicable disease risk factors (Figure 5H): Alcohol consumption (as a continuous variable) was associated with increased age gap for the Lehallier and Wang clocks only. BMI z-score was associated with increased age gap for the Oh and Wang clocks and with lower age gap for the Sathyan clock, while current smoking was associated with increased age gap for the Lehallier, Tanaka and Global clocks only. Among protective factors, including physical activity, healthy diet score and high education level, associations were generally in the expected direction, although only the association with physical activity (as a continuous variable) and lower age gap z-score for the Sathyan clocks were significant after FDR correction.

### Associations of organ specific proteomic age gaps with mortality and incident diseases

**Figure 6:**
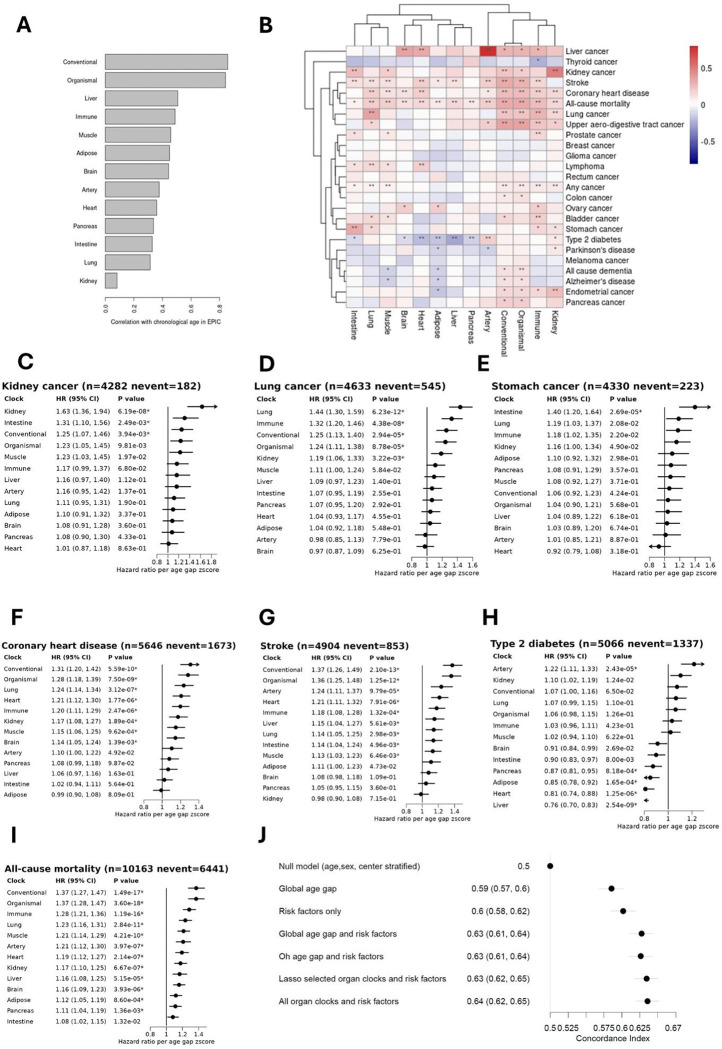
Associations of organ specific proteomic age gaps with mortality and incident diseases. All models generated using weights from study of Oh et al. Here were refer to the Oh clock, as previously presented, as “conventional”. A) Pearson’s correlations with chronological age in all EPIC samples B) Clustered heatmap showing risk factor-adjusted associations (log hazards per age gap z-score) for all -cause mortality and 25 incident diseases. * p<0.05; ** FDR-adjusted p <0.05. C-I): Hazard ratios from risk factor adjusted models per organ specific proteomic age gap z-score with C) kidney cancer, D) lung cancer, E) Stomach cancer, F) CHD, G) Stoke, H) type 3 Diabetes and I) All-cause mortality. Models stratified by study centre, sex, and five-year age group and adjusted for education level, smoking status, alcohol consumption, BMI, healthy diet score and physical activity. J) Comparison of discriminatory power showing concordance index for various models for prediction of mortality. All error bars show 95% confidence intervals.

Organ specific clocks were calculated using the weights generated by the study of Oh et al.^13^ trained in the same population as Oh clock (Figure 2). In the following section we have included the Oh clock as a comparison, referred to as a “Conventional” clock. All clock predicted ages were significantly associated with chronological age (Figure 6A). Conventional and organismal age gaps were very strongly correlated with each other (r=0.97), while other correlations between the organ-specific age gaps were weak to moderate, ranging from 0.08 to 0.46 (Figure S10).

After correction for FDR, the immune clock was positively associated with the most endpoints with eight significant associations, followed by the organismal age and conventional age gaps with seven associations, lung and kidney age gaps with six each, and the heart age gaps with five (Figure 6b). In some cases, organ -specific clocks showed stronger associations with diseases related to their organ such as for the kidney age gap and kidney cancer (HR: 1.63 (95% CI: 1.36–1.94, Figure 6C), lung age gap and lung cancer (HR: 1.44 (95% CI: 1.30–1.59, Figure 6D), and intestine age gap and stomach cancer (HR: 1.40 (95% CI: 1.20–1.64, Figure 6E). The heart age gap was most strongly associated with coronary heart disease (HR: 1.21(95% CI: 1.12–1.30), after the conventional, organismal and lung clocks (Figure 6F), while the artery clock was most strongly associated organ specific clock with stroke (HR: 1.24 (95% CI: 1.11–1.37, Figure 6G). The brain age clock was not associated with any of the neurodegenerative diseases. While there was a nominally significant association between the kidney clock and type 2 diabetes, type 2 diabetes was negatively associated with multiple organ clocks including the pancreas age gap. All but the intestine age gaps were associated with mortality (Figure 6I). We observed that the kidney clock clustered most closely with the immune, conventional and organismal clocks (Figure 6b).

The concordance index of a model predictive of all-cause mortality containing standard risk factors (education level, smoking status, alcohol consumption, physical activity, BMI and healthy diet index) was 0.60 (95% CI: 0.58–0.62), similar to a model containing only the Global age gap (0.59, 95% CI: 0.57–0.60). The concordance index improved to 0.63 (95% CI: 0.61–0.64), when risk factors and the Global age gap were combined. A parsimonious model of all-cause mortality was selected using lasso penalization and included standard risk factors and the organismal, artery, immune, heart, kidney, lung and muscle age gaps (model coefficients shown in supplementary figure S11). The concordance index of this model was 0.63 (95% CI: 0.62–0.65) and very similar to both the combined conventional Oh age gap and risk factor model, and to a model containing all organismal and organ specific age gaps and risk factors (Figure 6J).

## Discussion

In this large pan-European cohort with up to 28 years of follow-up, we conducted an extensive assessment of proteomic age gaps as markers for accelerated aging in relation to mortality and 24 incident diseases. We have shown that Somascan-based proteomic age gaps are associated with all-cause mortality risk across all proteomic clocks tested, indicating their utility as biological age metrics across independent populations. We found that the Global age gap, an unweighted average of age gaps derived from five previously developed clocks, was more strongly associated with mortality and many incident diseases than the individual clock estimates. The Global age gap was associated with age-related diseases including cardiovascular diseases, dementia, and cancers of the liver, upper aero-digestive tract, lung, and kidney. We observed a significant inverse association between the healthy lifestyle index and the Global age gap, suggesting that improving health behaviours may improve ageing trajectories. We found that proteomic age provided similar performance in predicting mortality to classical lifestyle risk factors, and that predictive power was further improved when risk factors and proteomic age gaps were combined, indicating that proteomic age provides additional information on mortality risk. We further observed that cancers of the kidney, stomach, and lungs were most strongly predicted by the proteomic age gaps in related organs.

Each additional year of Global age gap was associated with a 13% increase in mortality risk. Based on a conversion formula ^17^ derived from UK lifetables, we estimate that this is equivalent to approximately 6 months of life lost for every year of additional age gap. This is broadly in line with previous studies: the Tanaka clock was associated with a 3% increased risk of all-cause mortality per year of age gap in the Italian InCHIANTI study ^14^. Each year of the Sathyan clock was associated with a 12% increased risk of all-cause mortality in older adults of the US LonGenity study, in a model adjusted for chronological age and a frailty index ^11^. In the same LonGenity population, the Oh clock was associated a 50% increased risk of all-cause mortality, per SD (approximately 4 years) in age gap ^13^. The Wang clock was associated with a 38% increased risk of all-cause mortality among middle-aged participants in the US ARIC study per SD (approximately 3 years) in age gap ^12^. Associations were stronger using a “late-life” trained clock among older ARIC participants ^12^. The Argentieri clock, trained on Olink data in the UK Biobank was associated with an 8% increased risk of all-cause mortality per year of additional proteomic age ^7^. These studies demonstrate the consistency of effects observed across differently trained proteomic clocks.

The ageing process is theorized to include both intrinsic ageing, which results from accumulating damage from basic biological processes (i.e., the Hallmarks of ageing^1^) and occurs in the whole population as a function of time, and age-related, but avoidable disease processes. Since these clocks have been trained directly on chronological age among mainly healthy population samples including younger disease-free participants, it is likely they are mainly capturing a high-level summary of multiple intrinsic ageing processes ^18^. Although only a limited number of proteins were common across all conventional clocks, the moderate correlations and similar disease associations across the clocks suggest that they provide similar summary measures. “Second generation” clocks trained directly on time to death, rather than chronological age likely capture both intrinsic ageing and age-related disease processes. Indeed, a second-generation clock trained on mortality in UK Biobank Olink data by Goeminne et al ^8^. was associated with an almost three times greater mortality risk in out of fold predictions, compared to a “first generation” clock trained on chronological age in the same study.

We observed a significant correlation between the strength of age gap associations and the age-relatedness of diseases, derived from incidence data from UK national electronic health records. This adds further weight to both the validity of the use of proteomic clocks to assess biological age and to the Geroscience hypothesis that proposes biological ageing itself as a root cause of the development of many age-related diseases. Significant associations were observed with diseases such as dementia, cardiovascular disease and some cancers that show a similar pattern of incidence, characterized by an exponential increase in disease onset with age. Associations were not significant with diseases that show a more gradual relationship between disease onset and age or, like Hodgkin’s lymphoma, do not appear age-related. Argentieri et al. similarly reported associations with an Olink-based clock and age-related diseases in UK Biobank including stroke, heart disease, and dementia and others not investigated here such as chronic kidney disease, emphysema and osteoporosis ^7^. Among cancers, they also reported significant associations for esophageal and lung cancers, but unlike this study significant associations were also observed for non-Hodgkin lymphoma and prostate cancer. They did not observe significant associations with breast, colorectal, ovarian and liver cancers ^7^. In this study, which had almost twice as many cases as UK Biobank, we observed a highly significant association with liver cancer risk. Associations remained stable after exclusion of cases occurring within five years of proteomic age assessment.

As measured by the Global proteomic age, we observed that heavy smokers were on average 14 months biologically older than never-smokers, heavy drinkers were around seven months biologically older than non- or very light drinkers, and the least physically active were around three months biologically older than the most physically active. These associations suggest that a healthier lifestyle may slow intrinsic biological ageing, thereby reducing the risk of multiple age-related diseases in later life. However, in contrast to the relative consistency across conventional clocks in their associations with disease, associations with BMI, alcohol use and smoking were inconsistent across clocks. Inconsistent or relatively small associations with risk factors have been reported for other first-generation clocks ^19–21^ and may relate to differences in training population and how risk factors are distributed with age. For instance, BMI was positively associated with the Wang clock that was trained in middle-aged participants but negatively associated with the Sathyan clock that was trained in older participants. Changes in adiposity are a well-established part of the ageing phenotype, with increases generally observed as people enter middle age, followed by decreases in late life ^22^. Therefore, the Wang are Sathyan clock likely capture the proteomic profile of greater and lower adiposity respectively, in addition to biological age processes. The Global clock appears to ameliorate the influence of the training populations, and potentially also statistical error, through averaging age predictions produced in different populations. This is evidenced by slightly larger and more precise associations with mortality and more coherent associations with risk factors. As standardized proteomic data becomes more widely available around the world, future studies should train clocks in diverse populations, which may be further combined to provide more precise biological age estimates.

Organ specific clocks represent an advancement biological age assessment as they recognize that organ systems age at different rates within individuals ^23^. We observed some specificity in organ ageing and disease, in particular for kidney, lung, and stomach cancers, but also generalized effects, with organ age gaps affecting risk of disease across multiple organs. Broadly similar findings were recently reported in the UK Whitehall II cohort ^24^. For instance, while heart and artery ageing were among the strongest predictors of cardiovascular diseases, ageing across multiple organs contributed to risk of these outcomes. Immune system ageing predicted the most numbers of diseases emphasizing the central importance of inflammatory processes in ageing and chronic disease ^25^. Despite a weak association with chronological age within the EPIC study, the kidney clock was associated with six health endpoints and clustered together with the immune, conventional and organismal clocks, suggesting it is also capturing more systematic ageing processes. Indeed, the globular filtration rate, a marker of kidney function, is recognized as an effective marker of functional ageing ^26^. Although brain age has been associated with Alzheimer’s disease in cross-sectional analysis^13^ , the brain age gap did not predict risk of neurodegenerative outcomes here or in the Whitehall study over around 20 years of follow-up ^24^. It is likely that in mid-life, systematic ageing is a stronger predictor of later brain ageing and neurodegeneration. All organ age gaps were predictive of all-cause mortality, although the strongest associations were observed for conventional and organismal age gaps which are measures of systematic ageing. When combined into a single model we found that the organismal, artery, immune, heart, kidney, lung and muscle age gaps all provided independent predictions of mortality. However, overall prediction of the combined model was not improved compared to models using only a conventional age gap, suggesting that conventional clocks are sufficient for capturing the contribution of biological ageing for mortality prediction.

Limitations of this study include the single baseline assessment of proteins, which provides only a snapshot of biological age, while the risk factor analysis was cross-sectional, using lifestyle information collected once at baseline. Exposure misclassification in risk factor assessments may have also contributed to some residual confounding. The sample is overwhelmingly of White ethnicity, and while generally reflective of source population at the time of baseline assessment, reduces generalizability to other ethnicities. However, the study has considerable strengths. Its large sample size, particularly in the context of proteomic studies, and the design as a population-based sample greatly increases its generalizability across the European population. The case-cohort approach provides an efficient use of study resources to assess many cases across multiple disease classes, increasing study power. Case ascertainment used multiple approaches reducing disease misclassification. Finally, the longitudinal design limits reverse causality and allows investigation of how ageing in midlife influences disease risk in later life.

In the most comprehensive study to date, we have shown that an accelerated aging phenotype captured by proteomic clocks is associated with multiple chronic diseases and mortality. In addition to facilitating aetiological investigations into variability in biological ageing rate, potential applications of these clocks include improvement of disease risk and prognostic prediction models, more accurate patient stratification in clinical trials, and as surrogate endpoints in interventions and pharmaceutical trials aimed at reducing age-related disease. While further work is required before their application in clinical settings, particularly regarding the stability of biological age assessment on an individual basis, proteomic clocks show great promise for further understanding the links between ageing and health.

## Online Methods

### Study population

The European Prospective Investigation into Cancer and Nutrition (EPIC) is a cohort comprised of 521,32 participants (70.1% female) who were recruited between 1992 and 2000 across ten European countries ^27^. A subset of these participants was selected into the EPIC-Somalogic study, that aims to discover novel biomarkers of cancer, diabetes, cardiovascular diseases, neurodegenerative diseases and mortality using the Somascan proteomics assay.

Participants from EPIC centers in Italy, Spain, the Netherlands and the United Kingdom, with available baseline plasma samples and baseline dietary and lifestyle data, aged between 35 and 75 and complete follow-up data were eligible for the study. (Figure 1). The study followed a case-cohort design, which is an efficient design for prospective evaluation of multiple endpoints ^28^. Participants were selected into a main subcohort, previously used for the EPIC-InterAct and EPIC-Heart studies ^29,30^. This main subcohort is a randomly selected, representative sample of participants in the participating EPIC centres, including incident disease cases and deaths. In addition, case sets were selected for incident disease events and deaths including cancers, cardiovascular diseases, type 2 diabetes, and neurodegenerative disease (all-cause dementia, Alzheimer’s disease, Parkinson’s disease). For the study of neurodegenerative disease, additional samples were added to both the case and subcohort sample from Heidelberg study centre in Germany (Fig 1). For the Alzheimer’s disease and dementia case-cohort study, only Spanish centers contributed cases alongside non-cases.

EPIC was approved by the Ethics Committee of the International Agency for Research on Cancer (IARC), Lyon, France, and local ethics committees of the study centres. All participants provided written informed consent for the collection, storage, and individual follow-up of their data.

### Case ascertainment

Incident disease cases, coded using the 10th revision of the WHO International Statistical Classification of Diseases (ICD-10)], were identified using multiple sources of evidence including self-report, linkage to primary-care registers, secondary-care registers, medication use (drug registers), hospital admissions and mortality data ^27 30 29^. Information from any follow-up visit or external evidence with a date later than the baseline visit was used. Incident neurodegenerative disease cases were first identified by record linkage with existing health databases and subsequently validated by re-review of medical records ^35^. Data on total and cause-specific mortality were collected through mortality registries or active follow-up and death-record collection ^27^

### Proteomic assessment

Blood was collected in plasma citrate tubes at the baseline clinical assessment and processed into plasma and stored in liquid nitrogen the IARC central biorepository, in Lyon, France. Selected samples were shipped to the SomaLogic laboratory in Boulder, CO, USA on dry ice.

Plasma samples were analyzed using the SomaScan ver 4.1 array which quantifies approximately 7,000 proteins simultaneously ^31^. In brief, plasma samples were incubated with a mixture of modified aptamers to generate aptamer-protein complexes followed by several washing steps, elution of the fluorescently labelled aptamers from the target protein, and quantification on a DNA array (Agilent Technologies). Internal SomaLogic standardization and quality control (QC) procedures were then applied on the raw data, including hybridization normalization, intraplate median normalization, plate scaling and calibration using matrix matched calibrator controls. Adaptive Normalization by Maximum Likelihood (ANML) was applied using internal reference sets, to improve comparability with external datasets. Standard QC checks against triplicates on each plate and pre-defined acceptance criteria were applied, leaving 17,841 samples for which 7,285 aptamers (6,381 proteins) were available for analysis.

Samples flagged by SomaLogic for failing to meet standard acceptance criteria for ANML normalization were excluded (247 samples) ^32^. Additionally, we applied a further internal QC step to exclude 121 multivariate outliers, detected using unbiased PCA projection based on local outlier factor ^33^ and Tukey’s rule, using the *bigutilr* R package

### Proteomic age calculation

Proteomic ages were calculated using published weights from five studies, including Tanaka et al.^10^, Lehallier et al, ^15^, Sathyan et al. ^11^, Oh et al ^13^, and Wang et al. ^12^. We refer to these clocks as “conventional clocks” as they were trained using all available aptamers and we refer to each clock by the first author of the original study. For the Wang clock we used the version trained in midlife participants as closest to the age range of EPIC participants and to provide contrast with other clocks trained in older populations. Prior to calculation, protein intensity values were “lifted” to those provided with the v4 Somalogic assay using the multiplication scaling factors provided by Somlogic within the *lift_adat* within the *SomaDataIO* R package. Proteomic data was transformed and or scaled as specified in each publication, for appropriate clock calculation.

Proteomic clocks, including organismal, and organ specific proteomic clocks, developed by Oh et al were calculated using the python organage package (https://github.com/hamiltonoh/organage). No other studies have currently trained organ-specific clocks on SomaScan data. The organ-specific clocks were trained using proteins putatively annotated to their organ sources, based on differences between organs in gene expression levels using the GTEX database ^34^. In addition, an organismal clock was trained based using only proteins which were non-specific to a particular organ^13^.

To maintain consistency with previous approaches, the “Age gap” was calculated as described by Oh et al. ^13^ by fitting a local regression, that allows for non-linearity between predicted and chronological age using the *lowess* function in R for all clocks (with fraction parameter set to 2/3). Individual sample age gaps were then calculated as the difference between predicted age and the lowess regression estimate. The age gaps can be interpreted as the biological age component of proteomic predicted age, and are expressed as years of higher or lower proteomic age relative to one chronological age. The Age gaps were then z-scored for each clock, which was used as the primary exposure in all association analyses allowing direct comparison between the clocks, which may have differing variability.

Additionally, we calculated a “Global” clock age as the mean of the predicted ages from the Tanaka, Lehallier, Sathyan, Oh, and Wang clocks. The Global age gap was similarly calculated as the mean of these five conventional clock age gaps, and subsequently scaled to provide a z-score.

### Covariates

BMI (kg/m^2^) was derived from measured height and weight in all centers, except Oxford where it was self-reported^27^ . A validated index capturing all physical activity domains (Cambridge Index) was computed from physical activity during recreational activities and at work ^36^. Diet, including alcohol intake, was assessed using validated country- or center-specific dietary questionnaires designed to capture habitual consumption over the year preceding the study recruitment ^27^. A healthy diet score was derived from six dietary factors as previously described ^37^. Information on smoking status, education attainment, and menopausal status in women was obtained using lifestyle questionnaires ^27^. An overall healthy lifestyle index was determined by assigning scores of 0 to 4 to five lifestyle risk factors (BMI, smoking, alcohol use, diet, and physical activity) for which a higher point value indicates a healthier behavior, as previously described^37^

### Statistical analysis

Using the case-cohort design, including the subcohort and the respective mortality or disease samples, we tested associations of baseline Age gap z-scores against 25 incident disease endpoints. We fitted Cox proportion hazards regression models, using the *survival* R package with age as the underlying time scale, and stratified by sex, center, and 5-year age groups. To account for over representation of cases compared to the original cohort, we applied Prentice-weighting to cases outside the subcohort to provide unbiased inferences ^38^. Prevalent disease cases had been excluded already, except for 150 diabetics, who were removed for the analysis of incident type 2 diabetes.

We present both base models (stratified by sex, center, and 5-year age groups) and risk adjusted models that additionally included smoking status (never/former/current), alcohol consumption (grams/day), BMI (continuous score), healthy diet score (categorized into five quintiles), physical activity (metabolic equivalents of tasks (METs) and education level (None, Primary school completed, Technical/professional school, Secondary school, and Longer education (including University degree). Covariates were chosen *a priori* based on their well-established associations with mortality risk and data availability across all participants. The same adjustment set was used across all outcomes to maintain comparability. To maintain sample size across base and risk factor adjusted models, missing covariates (less than 6%) were imputed using a single imputation based on 50 iterations using the *mice* package in R for all samples. In addition to the covariates used in the risk factor models, covariates including sex, age, centre, main subcohort membership, healthy lifestyle index, and Mediterranean diet score were used as predictors in the imputation models.

Clustered heatmaps were drawn using the *pheatmap* package in R to display associations (log hazard ratios) across clocks/diseases. Clustering was performed using complete linkage hierarchical clustering. To account for multiple testing across the clocks and disease endpoints, we applied a 5% False discovery rate (FDR) correction, using Benjamini & Hochberg method (1995). Calculation of the FDR thresholds was conducted separately for the analysis of conventional and organ specific clocks.

We extracted information on the relationship between disease incidence and chronological age from the study Kuan et al. ^16^ which used the Clinical Practice Research Datalink, an electronic health record dataset from over 3 million individuals from across England. Specifically, for each disease we extracted the age-dependent component (β, the rate of disease onset increase over age) from Gompertz-Makeham models fitted by Kuan et al. A higher rate indicates a closer relationship between disease onset increase with age and implies the disease is more likely to be age-related. Across 22 diseases that could be matched across studies, we conducted Spearman’s non-parametric correlation to test the strength and significance of the relationship between the β coefficients from Kuan et al with log hazards from our risk-factor adjusted models with the proteomic age gap z-scores. We also classified the diseases in EPIC according to the age clusters identified by Kuan et al. The diseases assessed in EPIC included those in clusters 1 and 3, for which disease onset increases exponentially with age, those in cluster 4, for which disease onset increases more gradually with age, and one disease (Hodgkin’s lymphoma) in age cluster 5, which is not considered age related at all.

In sensitivity analyses, to understand the potential impact of early pathology on age gap and disease and mortality associations, we tested associations in the risk factor-adjusted models between the Global proteomic age gap and disease endpoints, restricting to events that occurred only after two years and after five years since recruitment. To explore the impact of smoking on associations we similarly restricted analyses to never smokers.

We examined associations between the quintiles of healthy lifestyle index and the five healthy life stye index components with the Global age gap z-score (as the dependent variable), using separate linear regressions in main subcohort, adjusted for sex, age, and study centre. All these risk factors were coded into five categories to aid comparison, using the least healthy category as the comparator.

We further examined the healthy lifestyle index as continuous score for all conventional clocks, adjusted for sex, age, and study centre. To examine the independent role of individual risk factors and examine consistency across clocks, we additionally fitted a linear model including sex, age, study centre, smoking status (current smoker versus never/former smoker), alcohol consumption (grams/day, scaled), BMI (continuous score, scaled), healthy diet score (continuous score, scaled), physical activity (metabolic equivalent of tasks, (METs), scaled) and education level (longer versus less education) for each conventional age gap z score. Risk factor coefficients from this mutually adjusted model were presented on a heatmap.

We used Lasso penalized cox regression to select organ specific clocks into combined model of all-cause mortality, using the *glmnet* R package. 5 fold cross-validation was performed to tune the lambda hyperparameter for optimum prediction. Selected organ age gaps were then used to fit a Cox model of mortality, including risk factors, stratified by sex, center, and 5-year age groups and Prentice weighted as previously described. Discriminatory power of different models for mortality (using risk factors only, Global age gap only, combined age gaps and risk factor models, and using all available organ specific and organismal age gaps) were compared using Harrell’s concordance index.

All analyses were performed in R ver. 4.3.1.

## Supplementary Material

This is a list of supplementary files associated with this preprint. Click to download.

EpicProteomicsbiomarkersofageingsupplementarytables.docx

EpicProteomicsbiomarkersofageingsupplementaryfigures.docx
